# Understanding Nursing Workflow for Inpatient Education Delivery: Time and Motion Study

**DOI:** 10.2196/15658

**Published:** 2019-11-01

**Authors:** Kelley M Baker, Michelle F Magee, Kelly M Smith

**Affiliations:** 1 MedStar Institute for Quality and Safety Columbia, MD United States; 2 MedStar Health Research Institute Hyattsville, MD United States; 3 MedStar Diabetes Institute Washington, DC United States; 4 School of Medicine and Healthcare Sciences Georgetown University Washington, DC United States

**Keywords:** nursing, workflow, time and motion studies, patient education, type 2 diabetes mellitus

## Abstract

**Background:**

Diabetes self-management education and support improves diabetes-related outcomes, but many persons living with diabetes do not receive this. Adults with diabetes have high hospitalization rates, so hospital stays may present an opportunity for diabetes education. Nurses, supported by patient care technicians, are typically responsible for delivering patient education but often do not have time. Using technology to support education delivery in the hospital is one potentially important solution.

**Objective:**

The aim of this study was to evaluate nurse and patient care technician workflow to identify opportunities for providing education. The results informed implementation of a diabetes education program on a tablet computer in the hospital setting within existing nursing workflow with existing staff.

**Methods:**

We conducted a time and motion study of nurses and patient care technicians on three medical-surgical units of a large urban tertiary care hospital. Five trained observers conducted observations in 2-hour blocks. During each observation, a single observer observed a single nurse or patient care technician and recorded the tasks, locations, and their durations using a Web-based time and motion data collection tool. Percentage of time spent on a task and in a location and mean duration of task and location sessions were calculated. In addition, the number of tasks and locations per hour, number of patient rooms visited per hour, and mean time between visits to a given patient room were determined.

**Results:**

Nurses spent approximately one-third of their time in direct patient care and much of their time (60%) on the unit but not in a patient room. Compared with nurses, patient care technicians spent a significantly greater percentage of time in direct patient care (42%; *P*=.001). Nurses averaged 16.2 tasks per hour, while patient care technicians averaged 18.2. The mean length of a direct patient care session was 3:42 minutes for nurses and 3:02 minutes for patient care technicians. For nurses, 56% of task durations were 2 minutes or less, and 38% were one minute or less. For patient care technicians, 62% were 2 minutes or less, and 44% were 1 minute or less. Nurses visited 5.3 and patient care technicians 9.4 patient rooms per hour. The mean time between visits to a given room was 37:15 minutes for nurses and 33:28 minutes for patient care technicians.

**Conclusions:**

The workflow of nurses and patient care technicians, constantly in and out of patient rooms, suggests an opportunity for delivering a tablet to the patient bedside. The average time between visits to a given room is consistent with bringing the tablet to a patient in one visit and retrieving it at the next. However, the relatively short duration of direct patient care sessions could potentially limit the ability of nurses and patient care technicians to spend much time with each patient on instruction in the technology platform or the content.

## Introduction

Persons living with chronic, complex medical conditions, including diabetes mellitus, must learn to self-manage their condition to enable optimal outcomes. Diabetes self-management education and support (DSMES) improves diabetes-related outcomes including glycemic control, risk of complications, and use of hospital and emergency room services [1-8]. Despite demonstrated efficacy of DSMES, as recently as 2015 almost half of people diagnosed with diabetes had never received diabetes self-management education [9]. In addition, less than 7% of patients with private insurance receive DSMES during the first year after diagnosis [10].

Strategies are needed to expand the reach of DSMES among the over 30 million persons living with diabetes in the United States [11]. DSMES is typically provided in the ambulatory practice setting in classes or via individual visits with a diabetes educator or nutritionist [12]. Adults with diabetes have high hospitalization rates for both diabetes-related and nonrelated diagnoses and high rates of 30-day readmissions when compared with persons without diabetes [13]. Therefore, hospital admissions present a critical opportunity not only for appropriate diagnosis and medical treatment but also for providing education to persons with diabetes.

Nurses, supported by patient care technicians (PCTs), provide much of inpatient care and are typically responsible for delivering patient education, including diabetes self-management education, at the bedside prior to discharge. Often diabetes specialty teams are not available, or such teams cannot meet the demand to teach all persons with diabetes. Integrating education into nursing unit workflow can present challenges. In the current health care environment, ever-increasing nursing staff workload and shortening lengths of stay impact the amount of time nurses and unit staff have available for patient care activities, including providing education [14-15]. In a survey of almost 3000 nurses on general medical-surgical units, 52% reported not having time to provide needed patient education on their last shift [16]. The use of technology to support education delivery in the hospital is one potential solution to these challenges. There is evidence that patients are willing to use tablet-based education programs and these programs can be effective for inpatient education [17-20].

Diabetes to Go is a diabetes education program that can be delivered to patients on a tablet computer via Web access [21]. The program provides diabetes survival skills education and consists of a 15-question validated knowledge test and short videos (most less than 3 minutes). It was designed to be used independently by the patient, and the full program takes 20 to 30 minutes to complete. The aim of this study was to evaluate nurse and PCT workflow, where workflow is defined as the frequency, duration, and pattern of activities, to identify opportunities for providing education. The results of this study were used to inform the design of implementing the Diabetes to Go intervention pragmatically in the hospital setting within existing nursing workflow with existing unit staff and minimal impact on workload.

## Methods

### Study Design

We conducted a time and motion study of nurses and PCTs on medical-surgical units of a large urban tertiary care hospital. A time and motion study is a quantitative data collection method where an observer continuously records the actions of a subject and, more specifically, the time and movements required to complete those actions [22]. Time and motion studies are often used to understand workflow to identify process efficiencies and improvements [22].

From May to July 2017, five trained observers conducted time and motion observations in 2-hour time blocks. The observers were all members of the research team; one observer was a medical assistant, while the others were bachelors- or masters-trained research assistants or coordinators without any clinical experience. During each 2-hour observation block, one observer shadowed a nurse and one observer shadowed a PCT, and each recorded the tasks, locations, and their durations. The 2-hour observation blocks were distributed across Monday through Saturday, from 10 am to 7 pm, which were considered by nursing to be the days and times most likely for education to be provided. Observations were scheduled for a specific date, time block, unit, and role (nurse or PCT). Two observers reported to the assigned unit at the assigned date and time and worked with the unit manager to find staff participants (one nurse and one PCT) who were willing to be observed by the study team. After the observers identified whom they would observe, they continuously recorded the nurse or PCT tasks and locations for 2 hours. The observers took a 1-hour break and then returned to the unit for an additional 2-hour observation period. Participants were not observed off the unit, as it was not relevant to the study and usually represented a personal break for the participant.

### Setting and Participants

The research was conducted on three medical-surgical units within a 912-bed tertiary care medical center in Washington, DC. Two of the units are standard medical-surgical units. The third unit is a cardiac care unit, where most patients are recovering from cardiac surgery. We selected the units based on their high census numbers for adult patients with type 2 diabetes. Participants were nurses and PCTs who provided verbal consent to be observed as they performed their typical duties. To assure employee privacy and confidentiality, we did not collect descriptive information from the participants being observed, and the observation data could not be directly linked back to any individual. The MedStar Health Research Institute institutional review board approved the research.

### Data Collection

Initial task and location categorizations were developed based on the Omaha System nursing taxonomy [23] and a time and motion study of nurses by Schenk et al [24]. The task categories included teaching and guidance, treatment and procedures, case management, surveillance, electronic health record interaction, reading, communicating, and walking between locations. Location categories included patient room, hallway, team area at a computer, team area but not at a computer, medication room, supply room, nutrition room, and off unit. Early pilot testing demonstrated that it was difficult for observers, particularly nonclinical observers, to reliably discern the more specific task categorizations without disrupting the participants to ask what they were doing. In addition, when nurses are performing direct patient care, they frequently multitask (for example, educating the patient about a medication while administering the medication) and task switch, with blurred lines between when one task ends and another begins. Because the purpose of this study was to identify opportunities to provide education, we determined that it was important to know when the nurse or PCT entered a patient room, how long he or she engaged in direct patient care before leaving the room, and when he or she returned to the room, as these would affect the ability to provide education. The specific tasks of direct patient care and specific locations when staff were not in the patient room were deemed not important for the study. Therefore, task and location categories were simplified, and four task categories and three location categories were defined for use during the observations (Textbox 1).

**Figure 1:**
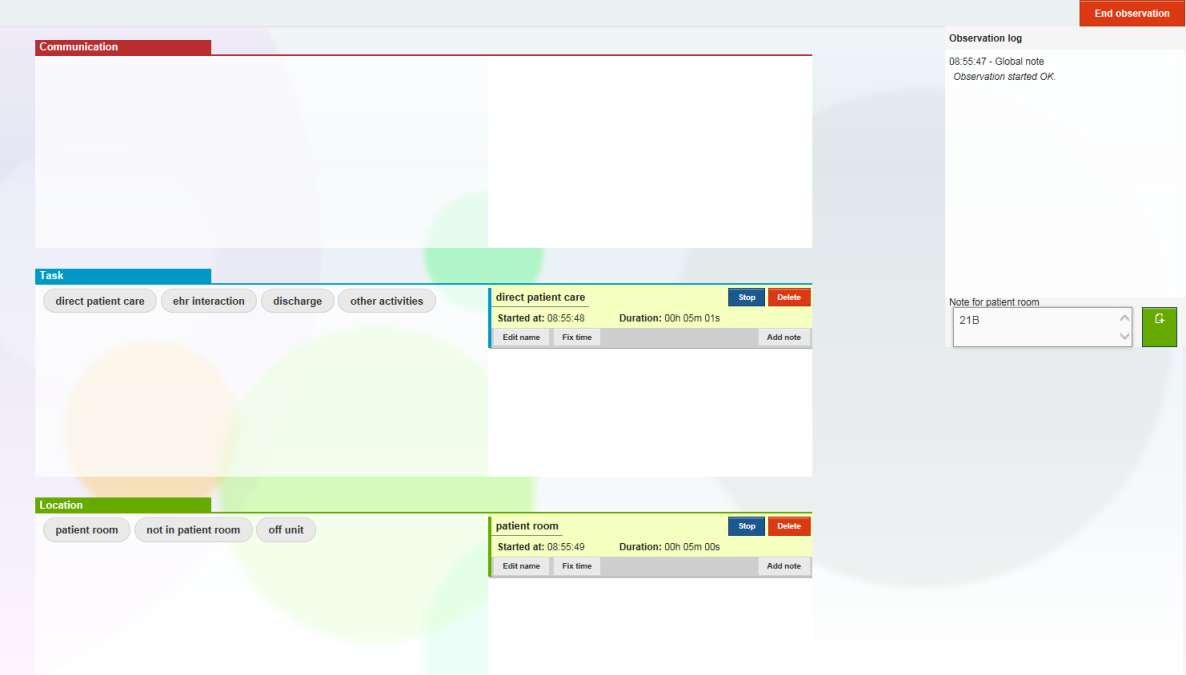
TimeCaT interface for study.

To record observations, observers used a tablet computer to access a Web-based time and motion data collection tool, TimeCaT [25]. The TimeCaT interface allows the user to specify co-occurring task, location, and communication (Figure 1). Note that the TimeCaT communication domain was not used in this study. Instead, communication was classified as other activities in the task domain. When the user selects a new task or location, TimeCaT timestamps the data entry. The user can also enter notes attached to each data entry. The study team used this feature to record the patient’s room number when in a patient room was selected as the location.

Prior to the start of data collection, the observers attended a 2-hour training to ensure common understanding of the study and observation procedures. The training included instruction on the purpose of the study, observation procedures, definition of each task and location category, and use of the TimeCaT data collection tool. After the classroom training, multiple paired observations were conducted to confirm interobserver reliability. TimeCaT includes a feature to calculate the kappa coefficient for paired observers. Through the commutative property, all observers were confirmed to be interreliable. Kappa values for consistency in naming each task and location ranged from 0.77 to 1.00, and kappa values for consistency in the proportion of time within each task and location ranged from 0.90 to 0.99, which indicated excellent agreement [26].

**Study task and location category definitions.**

Task domain:

- Direct patient care: any in-person interaction with the patient
- Discharge activities: a specific type of direct patient care; any in-person interaction with the patient where discharge was specifically discussed
- Charting: interaction with the electronic health record
- Other activities: any task that did not fit one of the three previous categories, including, for example, retrieval of medications or supplies, communication with other health care team members, and travel between patients

Location domain:

- In a patient room: in a room occupied by a patient
- Not in a patient room: outside of a patient room but on the unit, including, for example, medication room, supply room, nurses’ station, and hallway outside the patient rooms
- Off unit: not on the unit

### Data Analysis

Data collected in TimeCat was exported to an Excel (Microsoft Office 365 ProPlus, Microsoft Corp) spreadsheet for manipulation, and statistical analyses were conducted using the statistics program SPSS Statistics version 19 (IBM Corp). Percentage of time spent on a task and in a location and mean duration of task and location sessions were calculated and compared across roles (nurse vs PCT), day of the week, time of day, and unit. Analysis of variance was used to test for significance. In addition, the number of sessions per hour on a given task or in a given location, number of patient rooms visited per hour, and mean time between visits to a given patient room were determined.

## Results

### Observation Summary

The study team conducted 92 2-hour observation sessions, resulting in 182.4 hours of observations. There were 46 sessions for 91.4 hours with nurses and 46 sessions for 91 hours with PCTs. The observations were conducted Monday through Saturday, between 10 am and 7 pm, on three units, including two general medical-surgical units and one cardiac care unit (Table 1).

In many cases, the same participant was observed for two consecutive 2-hour blocks. It is possible the same participant was observed on multiple days, but we did not collect identifying information from the participants, and thus cannot confirm that possibility.

### Percentage of Time on Task and in Location

Nurses spent on average approximately one-third (32%) of their time in direct patient care, including discharge-related activities completed with a patient. One-quarter (25%) of nurses’ time on average was spent charting, and the remainder was spent on other activities such as retrieval of medications or supplies, communication with other health care team members, and travel between patients (Table 2). Nurses spent the bulk of their time (60%) on the unit but not in a patient room (Table 3); this included the nurses’ station, hallway outside the patient room where nurses do much of their electronic health record charting on a computer workstation on wheels, medication room, and supply room. Compared with nurses, PCTs spent, on average, a significantly greater percentage of their time in direct patient care (42%; *P*=.001) and on other activities (54%, vs 43%; *P*=.003), while nurses spent more time charting (Table 2). Compared with nurses, PCTs spent, on average, a significantly greater percentage of their time in a patient room (47% vs 33%; *P*<.001; Table 3).

**Table 1:** Study participants by role and unit.

Task	Nurses observed	PCTs^a^ observed
Medical-surgical unit 1	14	14
Medical-surgical unit 2	15	15
Cardiac care unit	17	17

^a^PCT: patient care technician.

**Table 2:** Percentage of time spent by task.

Task	Nurses, mean (SD)	PCTs^a^, mean (SD)	*P* value
Charting	25 (14.3)	4 (7.1)	<.001
Direct patient care	31 (16.6)	42 (14.8)	.001
Discharge activities	1 (2.5)	—^b^	.14
Other activities	43 (18.3)	54 (15.2)	.003

^a^PCT: patient care technician.

^b^Not applicable.

**Table 3:** Percentage of time spent by location.

Location	Nurses, mean (SD)	PCTs^a^, mean (SD)	*P* value
In patient room	33 (15.7)	47 (16.8)	<.001
Not in patient room	60 (16.8)	42 (16.1)	<.001
Off unit	8 (12.9)	11 (14.5)	.24

^a^PCT: patient care technician.

### Task and Location Sessions

Nurses averaged 16.2 tasks per hour, which included 5.1 direct patient care tasks per hour. The mean length of a direct patient care session was 3:42 minutes, while charting sessions and discharge activities were slightly longer at 4:57 minutes and 4:28 minutes, respectively (Table 4). PCTs averaged 18.2 tasks per hour, of which 8.2 were direct patient care tasks. PCTs’ mean session length was 3:02 minutes for direct patient care, 3:22 minutes for charting, and 3:27 minutes for other activities (Table 4). It is important to note that these averages are the result of many short sessions and fewer, longer sessions. For nurses, 56.37% (836/1483) of task durations were 2 minutes or less, and 38.23% (567/1483) were 1 minute or less. An even greater percentage of PCT task durations were short, with 61.99% (1039/1676) being 2 minutes or less and 44.27% (742/1676) being 1 minute or less; 9.10% (135/1483) of nurse tasks and 6.68% (112/1676) of PCT tasks were longer than 10 minutes.

Nurses averaged 13.7 locations per hour, and PCTs averaged 19.6 locations per hour. Nurses spent an average of 3:41 minutes in a patient room whereas PCTs spent an average of 2:57 minutes in a patient room (*P*=.03; Table 5). Again, the location duration averages are the result of many short sessions and fewer, longer sessions. For nurses, 52.99% (683/1289) of location durations were 2 minutes or less, and 36.85% (475/1289) were 1 minute or less. For PCTs, 63.97% (1131/1768) of location durations were 2 minutes or less and 48.02% (849/1768) were 1 minute or less. For nurses, 11.64% (150/1289) of location durations were longer than 10 minutes, while for PCTs, 6.39% (113/1768) were longer than 10 minutes.

**Table 4:** Session duration on task.

Task	Nurses, mean^a^ (SD)	PCTs^b^, mean (SD)	*P* value
Charting	4:57 (6:44)	3:22 (5:24)	.06
Direct patient care	3:42 (5:05)	3:02 (4:12)	.07
Discharge activities	4:28 (6:59)	—^c^	.50
Other activities	3:12 (5:54)	3:27 (7:59)	.42

^a^Mean session durations reported in minutes and seconds.

^b^PCT: patient care technician.

^c^Not applicable.

**Table 5:** Session duration by location.

Task	Nurses, mean^a^ (SD)	PCTs^b^, mean (SD)	*P* value
In patient room	3:41 (4:58)	2:57 (4:12)	.03
Not in patient room	4:22 (7:12)	2:31 (5:37)	<.001
Off unit	15:20 (13:48)	17:42 (16:40)	.12

^a^Mean session durations reported in minutes and seconds.

^b^PCT: patient care technician.

### Room Visits

Nurses visited 5.3 (SD 2.2) and PCTs 9.4 (SD 4.0) patient rooms per hour. The mean time between nurse visits to a given room was 37:15 minutes and between PCT visits to a given room 33:28 minutes. In the 2-hour observation blocks, 36.7% (66/180) of rooms visited by a nurse being observed were only visited once by that nurse, and 42.6% (162/380) of rooms visited by a PCT being observed were only visited once by that PCT.

### Day of Week, Time of Day, and Unit Comparisons

Comparisons of measures across day of the week were conducted to determine if there were differences that might make one day better or worse than another for providing education. There were no significant differences across day of the week for nurses or PCTs for percentage of time spent on a task category or in a given location. In addition, there were no significant differences in the mean number of task or location sessions per hour. For nurses only, there was a statistically significant difference in session duration for other activities, with a high on Saturday of 5:20 minutes and a low on Tuesday of 2:23 minutes. This trend was not observed in PCTs.

We also compared measures across time of day to determine if there were differences that might indicate that a given time of day would be better or worse for providing education. To make this comparison, we grouped observations that started from 10 am to 12 pm as morning, observations that started from 1 pm to 3 pm as midday, and observations that started from 4 pm to 5 pm as late afternoon. Nurses spent a significantly greater percentage of their time off the unit during midday (observation blocks that started at 1 pm, 2 pm, or 3 pm). Off unit session duration was also significantly longer in this time block. In addition, nurses spent significantly more time charting in the midday time block. There were no other significant differences across time of day for nurses. For PCTs, the percentage of time spent charting and the mean session length for charting were significantly greater during the late afternoon observation blocks (starting at 4 pm or 5 pm). There were no other significant differences by time of day for PCTs.

Across the three study units, there were no significant differences in study metrics for nurses. PCTs on the cardiac care unit generally had more and shorter sessions than the PCTs on the two medical-surgical units.

## Discussion

### Principal Findings

This time and motion study of nurses and PCTs on medical-surgical units revealed important findings about staff workflow in an urban tertiary care hospital, specifically about the potential to support tablet-delivered bedside diabetes education. While providing patient education is a nursing responsibility, our prior research showed that PCTs were interested in contributing to patient education activities [27], so we also considered the workflow of PCTs. Nurses visited an average of 5.3 patient rooms per hour, while PCTs visited 9.4 patient rooms per hour. The workflow of nurses and PCTs, constantly in and out of patient rooms, suggests an opportunity for either a nurse or PCT to deliver a tablet to the patient bedside. In addition, the average time between visits to a given room is consistent with bringing the tablet to a patient in one visit and retrieving it at the next visit. The average time between nurse visits to the same patient room was 37:15 minutes and between PCT visits was 33:28 minutes. This time span would allow the patient sufficient time to engage with the education. To our knowledge, there are no other studies in the literature reporting a room visit analysis similar to that reported here (ie, time between visits to the same room). These findings add to the body of knowledge on nursing workflow on inpatient medical-surgical units and demonstrate the feasibility of a nurse or PCT completing an activity that requires them to visit a patient room initially and then return to the same patient room within a timeframe that is neither immediate nor as long as an hour.

It is possible then, within existing workflow, to drop off and pick up a tablet computer for diabetes education delivery. However, the relatively short duration of direct patient care sessions, at an average of 3:42 minutes for nurses and 3:02 minutes for PCTs, could potentially limit the ability of the nurses and PCTs to spend much time with each patient on instruction in use of the technology platform or in answering questions about the content. This suggests that it would be important for the patient to be able to engage with the education independently. We also found that some rooms were visited only once by the nurse or PCT being observed, but we believe that this may be an artifact of the 2-hour observation periods and the observation of a single care team member. When a room is initially visited late in a given observation period, a return visit would not necessarily be expected until after that observation period had ended. In addition, other care team members may have visited those rooms.

The finding that nurses spent approximately one-third of their time in a patient room in direct patient care is consistent with other studies in the literature, where time and motion studies report that nurses average 22% to 37% of their time in direct care activities [28-30] and 31% to 34% of their time in a patient room [24]. The average is somewhat higher for nurses on intensive care units at 41% to 50% [31-32].

Of perhaps greater relevance to capacity to deliver education is the length of time spent on individual tasks. We found that fully 38% of nursing tasks and 44% of PCT tasks were accomplished in less than one minute. The high percentage of short duration tasks indicates significant task switching and highlights the challenge of providing patients with effective in-person education or instruction in the use of the tablet computer within the current workflow. It is difficult to make comparisons between this study and other similar studies on duration of individual tasks and number of tasks per hour due to inconsistency in task definitions. In this study, we used four task categories, while other similar studies used, for example, 10 [28], 10 and 11 [33], 29 [34], and 41 [32] task categories, as dictated by the goals of the research. It is not surprising that a study with more specifically defined tasks would find more tasks per hour and tasks of shorter duration. In a study with more task categories, a participant might complete multiple individual tasks that would be classified as a single task of direct patient care in our study. For example, Cornell et al [33] reported more than 50% of tasks were completed in 30 seconds or less in an observation study of nurses on medical-surgical and pediatric oncology units that used 10 (medical-surgical) and 11 (pediatric oncology) task categories, and Douglas et al [32] found that nurses switched tasks an average of every 29 seconds in their study of adult and pediatric intensive care unit nurses where they used 41 task categories. Despite the differences in the number of task categories, these similar studies all conclude that nurses experience high levels of task switching and fragmented workflow [28,32-34].

Within the days and times of the study, there was no day of the week or time of day where nurses spend a greater percentage of their time in a patient room in direct patient care or have longer sessions in a patient room in direct patient care. Not surprisingly, nurses spent a significantly greater percentage of their time off the unit during midday, likely due to their lunch break. We conclude that, with the possible exception of midday, the data do not indicate that any day of the week or time of day, within the days and times observed, provides a better or worse opportunity for nurses and PCTs to deliver education.

There were several significant differences in nursing workflow compared to PCT workflow. These differences are due to the differences in responsibilities and patient load for the two groups. PCTs have little or no charting and discharge responsibilities. It is not surprising then that PCTs spend significantly more of their time in direct patient care; nurses spend a quarter of their time charting which leaves less time for direct patient care. In addition, on the study units, the PCTs are typically responsible for approximately twice as many patients as the nurses. It follows that PCTs would have more locations per hour and more tasks per hour as they divide their time among more patients. And while PCTs spend a greater portion of their time in direct patient care, the average duration of a direct patient care session is lower than a nursing direct patient care session. Overall, these results are consistent with either a nurse or PCT dropping off and picking up a tablet computer within their existing workflow.

### Limitations

There were several limitations to this study. First, although the units in the study are typical medical-surgical and cardiac care units, the study was conducted in a single hospital. While this served our study purpose of designing a process to implement a diabetes education intervention in that hospital, it potentially limits generalizability of the study outcomes. Second, participants were chosen based on their willingness to be observed, which may have introduced selection bias, and they were aware that they were being observed, which may have influenced their decisions on how and where to spend their time. Observers attempted to mitigate this by explaining that they were objectively recording what the participants were actually doing and not making subjective judgments about what participants should be doing. In addition, we did not attempt to assess how the nurses prioritized their time. We assumed that if a nurse was not in a patient room, he or she had a higher priority task outside the patient room and was not available to provide education. We also did not attempt to characterize the specific tasks done with the patient. We assumed that any visit to a patient room could potentially be used to deliver the education program but did not gather data to support this assumption.

### Conclusions

DSMES has been widely shown to be beneficial for persons with diabetes. In the hospital, nursing staff are responsible for providing patient education, but time and resource constraints often limit education delivery. This study generated data showing that nurses and PCTs make frequent short trips into patient rooms and constantly task switch. The data suggest that, within current workflow on hospital general medical-surgical nursing units, it would be feasible for nurses or PCTs to provide a technology-delivered diabetes education program to the bedside for patients to complete independently between staff visits to the room. Future research should pursue pragmatic implementation of delivering tablet-based patient education.

## Abbreviations

DSMES: diabetes self-management education and support
PCT: patient care technician
